# Do financial aspects affect care transitions in long-term care systems? A systematic review

**DOI:** 10.1186/s13690-022-00829-y

**Published:** 2022-03-23

**Authors:** Estera Wieczorek, Ewa Kocot, Silvia Evers, Christoph Sowada, Milena Pavlova

**Affiliations:** 1 Department of Health Economics and Social Security, Institute of Public Health, Faculty of Health Sciences, Jagiellonian University Collegium Medicum, Krakow, Poland; 2 Department of Health Services Research, Care and Public Health Research Institute (CAPHRI), Faculty of Health, Medicine and Life Sciences, Maastricht University, Maastricht, The Netherlands

**Keywords:** Transitional care, Care coordination, Care integration, Financing

## Abstract

**Background:**

Suboptimal care transitions of older adults may ultimately lead to worse quality of care and increased costs for the health and social care systems. Currently, policies and financing often focus on care in specific settings only, and neglect quality of care during transitions between these settings. Therefore, appropriate financing mechanisms and improved care coordination are necessary for effective care transitions. This study aims to review all available evidence on financial aspects that may have an impact on care transitions in LTC among older adults.

**Methods:**

This systematic review was performed as part of the European TRANS-SENIOR project. The databases Medline, EMBASE (Excerpta Medica Database) and CINAHL (Cumulated Index to Nursing and Allied Health Literature) were searched. Studies were included if they reported on organizational and financial aspects that affect care transitions in long-term care systems.

**Results:**

All publications included in this review (19 studies) focused specifically on financial incentives. We identified three types of financial incentives that may play a significant role in care transition, namely: reimbursement mechanism, reward, and penalty. The majority of the studies discussed the role of rewards, specifically pay for performance programs and their impact on care coordination. Furthermore, we found that the highest interest in financial incentives was in primary care settings.

**Conclusions:**

Overall, our results suggest that financial incentives are potentially powerful tools to improve care transition among older adults in long-term care systems and should be taken into consideration by policy-makers.

**Trial registration:**

A review protocol was developed and registered in the International Prospective Register of Systematic Reviews (PROSPERO) under identification number CRD42020162566.

**Supplementary Information:**

The online version contains supplementary material available at 10.1186/s13690-022-00829-y.

## Background

Care transitions are an integral part of a patient’s journey throughout a health care system [1]. Transitions of care can be defined as “a set of actions designed to ensure the coordination and continuity of health care as patients transfer between different locations or different levels of care within the same location. Representative locations include (but are not limited to) hospitals, sub-acute and post-acute nursing facilities, the patient’s home, primary and specialty care offices, and long-term care facilities” pp.556 [2]. In line with this definition, in this paper, we focus on transitions not only in the healthcare sector but also in the social care sector, as they seem equally important [1]. Thus, for the purpose of this study, we define the term “care transitions” as transitions happening in both, health and social sectors.

Care transitions are vulnerable exchange points and may result in negative clinical outcomes, preventable adverse events, and avoidable hospital readmissions. Suboptimal care transitions may ultimately lead to worse quality of care and increased costs for the health and social care systems, and therefore, their optimization is a policy priority [3]. Care transition is optimized by improving care for the patient and/or avoiding unnecessary care transitions. Suboptimal or fragmented care transitions may not only lead to unnecessarily high rates of health services use and health care spending, but they may also expose chronically ill people to lapses in quality and safety [1, 4]. Transitions between different care settings are recognized as high-risk scenarios for patient safety and should be avoided or optimized when possible [1]. Researchers seem to agree that older patients are particularly vulnerable to breakdowns in care and, therefore, may be the most in need of transitional care services [5, 6].

Several factors, such as inaccuracies in information exchange, ineffective planning or coordination of care between care providers and lack of follow-up, may affect the care transition of a patient and may either hinder or promote smooth travel across varied settings of care and among multiple providers [5, 7, 8]. Financial aspects play an essential role in care coordination and care transitions [9]. Currently, policies and financing often focus on care in specific settings only, and neglect quality of care during transitions between these settings [5, 10]. Therefore, appropriate financing mechanisms and improved care coordination are necessary for effective care transitions [9, 11]. A financing mechanism will be considered appropriate if it provides incentives for high-quality care and effective management of transitions for good clinical outcomes and reduction of avoidable health care costs [2, 12].

The expectations to improve quality of care and care transitions through financial incentives that affect providers’ behavior, are mainly drawn from general economics, e.g. the works of Keneth Arrow [13], the new institutional economics and principal-agent theory [14], and behavioral economics [15]. According to the principal-agent theory, for example, health care providers not only act for the benefit of the patient but also attempt to maximize their own benefits against the interests of patients [14]. This is particularly problematic when incentives lead to market failure. For example, fee-for-service payment creates strong provider incentives for higher volume, especially for services with higher profit margins per unit of service. Nevertheless, it does not necessarily encourage the provider to improve quality of care or reduce total treatment costs. Additionally, behavioral economics highlights the role of rewards and penalties among health care providers and how they may shape providers’ behavior. Overall, the effect of the financial incentives on quality of care depends on the nature of the incentive. Different financial incentives and their mechanisms are widely described in the literature [16]. For instance, physicians may have a very different response to general incentives (e.g. capitation) versus selective incentives (e.g. Pay for Performance (P4P) programs). A selective incentive is thought to be more powerful in motivating physician quality response on the specific dimension (e.g. care coordination). This is because selective incentive can target a specific domain of quality and general incentive does not [16].

To the best of our knowledge, no overview exists on financial aspects that affect care transition of older adults in long-term care (LTC) systems. Majority of available studies either focus solely on one specific financial aspect [17, 18] or do not focus on older adults but rather the general population [19]. Therefore, this study aims to review all available evidence on financial aspects that may have an impact on care transitions in LTC among older adults.

The aim of this paper is to identify financial aspects that affect the care transition of older adults in LTC systems. A secondary aim is to identify the settings in which these financial incentives have been applied and to synthesize their reported impact on care coordination. As it is difficult to define fixed boundaries for LTC and many activities in various parts of the health system may influence significantly care transitions of older people, some areas not obviously related to classical LTC users were included in the analysis, e.g. diabetic care, hypertension, coronary heart failure etc.

## Methods

For transparency in this systematic review, a review protocol was developed and registered in the International Prospective Register of Systematic Reviews (PROSPERO) under identification number CRD42020162566. We performed the overall search in a systematic way to minimize the potential bias. Specifically, the PRISMA-P (Preferred Reporting Items for Systematic Reviews and Meta-Analyses) guidelines were followed to design the search strategy [20]. The PRISMA checklist is provided in additional file (see Supplementary Table 1). This review was performed as part of the European TRANS-SENIOR project [21].

As mentioned earlier, this systematic review focused on financial aspects of care transitions. It is carried out parallel to another systematic review focused on the organizational aspect in care transitions, which is reported in the same review protocol as both reviews build on the same overall search (registered in PROSPERO). The objective of the overall search was to identify all studies that address the financial and/or organizational aspects of care transition in the LTC systems.

### Data Sources and Search Strategy

The overall literature search was conducted in Medline, Embase and CINAHL. The search strategy was developed by the research team in consultation with an academic health sciences librarian. Given the search objective, three components were used to build the search terms for the identification of key financial and organizational aspects affecting care transition in LTC systems. These components included: (1) old or geriatric or senior; (2) care transition or coordinated care or care continuity; (3) financing or organization. Moreover, different forms of the above words as well as relevant synonyms and subject heading terms appropriate for each database, were taken into account. All search terms can be found in Table 1.

**Table 1:** Search terms

Category 1	Category 2	Category 3
Elderly	Patient*	Financ*
Aged	Care*	Organi*
Aging	Clinical handover	Purchas*
Old	Coordinated care	Funding
Senior	Coordination of care	Provision
Geriatric	Continuity of care	Reimbursement
	Integrated care	

Patient* captures i.a.“patient handover”, “patient transfer”, “patient discharge” etc. Care* captures i.a. “care coordination”, “care continuity”, “care continuum” etc. Financ* captures “financing”, “financial” etc. Organi* captures i.a. “organizational”, “organizing”, “organization” etc. Purchas* captures “purchasing”, “purchase” etc.

The exact chain of keywords used for the different databases can be found in Appendix 1. The search was limited to literature published between March 2005 and March 2020 (the last 15 years). No geographical or language restrictions were implied.

### Eligibility Criteria

Our overall search included studies that focus on transitional care between the settings among older adults 60+. Sixty years of age was selected as an age describing “older adult” as suggested by the World Health Organization. No restrictions were placed on participants’ gender or other demographic characteristics. All primary epidemiological observational study designs (i.e., cross-sectional, cohort, case-control studies), ecological studies and experimental studies were eligible. Reviews, commentaries, editorials and other non-primary research articles were excluded. Inclusion and exclusion criteria applied in the overall search are described below. Studies were included if (a) they reported on financial and organizational aspects of care transition in the LTC systems, (b) reported on financial and organizational aspects of care transition at the macro-level, mainly focusing on transitions between different settings and not within the setting (c) and their focus was on older adults (60 years or older). Studies were also included if data stratification was performed for individuals aged 60+. Studies were excluded if (a) they reported on financial and organizational aspects of care transition at the micro-level, care transition within the setting, (b) focus of the study was on individuals younger than 60 years of age, (c) focus was on palliative, hospice or end-of-life care.

### Study Screening and Selection

All references identified by the overall search queries were downloaded in Mendeley and duplicates were removed. The selection process, based on the above inclusion and exclusion criteria, had three phases. First, a screening based on title and abstract was performed by the main researcher (E.W.) to identify potentially relevant studies, and 10% of the excluded papers were independently reviewed by the other four researchers (M.P., E.K., S.E., C.S.). This was followed by a second screening based on full text to confirm the relevance of the studies. Third, the reference lists of the selected studies were screened to check for additional studies. Any disagreement about the eligibility of studies was resolved through discussion and consensus among all co-authors, as recommended in the literature [20].

The selected publications were then classified into financing and organizational categories. Thus, in this review, we only included studies that touch upon the financing of care transition.

### Data Extraction

A data extraction form was developed and pre-tested. The extracted information included, among others: author, year of publication, type of study, research approach, data collection method, study group, type of financial mechanism, aim of the mechanism, target group, intervention setting and country, measurement, results related to the implementation of financial mechanism (if possible) and recommendations regarding the financial mechanism.

### Quality Assessment

The methodological quality and risk of bias of studies meeting inclusion criteria were rigorously appraised with the use of Quality Assessment Tool for Quantitative Studies developed by Effective Public Health Practice Project (EPHPP) [22] and Critical Appraisal Skills Programme (CASP) [23] for qualitative studies. Tool assessing quantitative studies led to an overall methodological rating of strong, moderate or weak in eight sections: selection bias, study design, confounders, blinding, data collection methods, withdrawals, intervention integrity. A rating was performed according to the guideline provided along with the tool [22]. Tool assessing qualitative studies included 10 questions referring to aspects such as validity of the study, results and usefulness of results. For each question, there were three possible answers: yes/no/can’t tell. If an answer was,yes”, one point was assigned, if the answer was,no” or,can’t tell’ a question received zero points. In total studies could score 10 points. Studies that scored less than 33% (3 points) of total points were rated as low quality studies. Studies that scored from 33 to 66% of total points were considered as of moderate quality. At last, studies that scored more than 66% (7 points) of total points were regarded as high quality studies. Studies with mixed methods were assessed with the use of both checklists.

### Data Synthesis

The method of directed (relational) content analysis by Hsieh and Shannon (2005) [24] was applied to perform the analysis of the publications. Within this approach, we identified the categories (themes) relevant to the review objective. The preliminary literature search provided guidance for initial codes. Thus, for the purpose of this review, the following themes were used: reimbursement mechanism, reward, penalty.

Based on these themes, the data extraction on financial aspects was performed using the data extraction form mentioned above. Review results are presented per themes in a narrative manner.

## Results

**Fig. 1:**
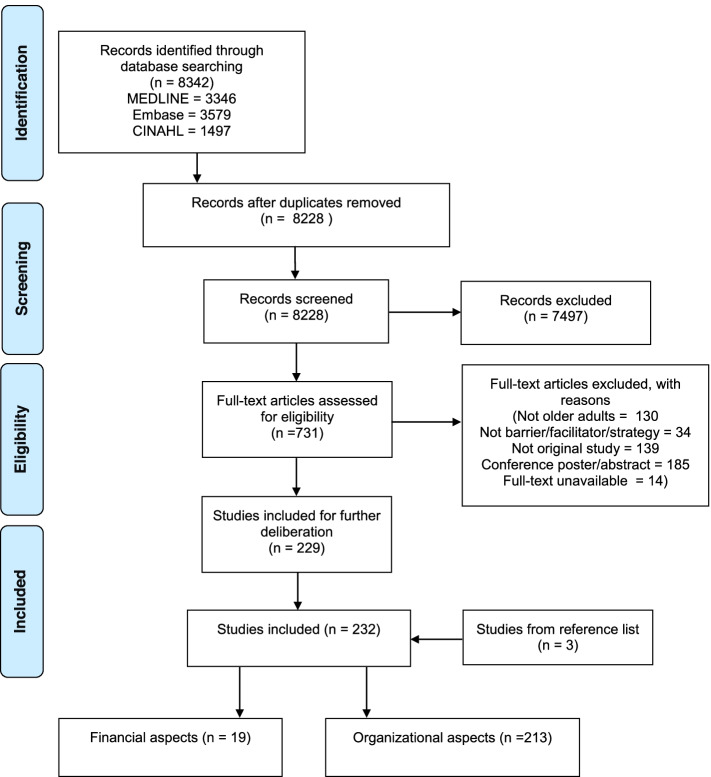
Stages of the selection process. Based on: Moher, D., Liberati, A., Tetzlaff, J., & Altman, D. (2009). Preferred Reporting Items for Systematic Reviews and Meta-Analyses: The PRISMA Statement. *Plos Medicine*, *6*(7). https://doi.org/doi:10.1371/journal.pmed.1000097 [25]

The overall search of the databases yielded 8342 publications. After removing duplicates, 8228 publications were included in the initial screening (see flowchart, Fig. 1). After reviewing the titles and abstracts, 7497 publications were excluded, as they did not meet the inclusion criteria. In total, 731 publications were included for the screening based on full text. The number of excluded full-text articles with reasons is presented in Fig. 1. Publications were then divided by topic: organizational and financial aspects. Ultimately, 19 records on financial aspects were included in this review.

### Study Characteristics

An overview of the characteristics of the studies included in this review, is presented in Table 2. The total number per category may exceed 19 as papers can be classified in multiple sub-categories. The majority of the publications have been published in the last 8 years (*n* = 12). The research approaches used by the researchers were quantitative (*n =* 12), qualitative (*n* = 5), mixed (*n* = 2). We identified studies with an explanatory aim (*n* = 15) and an exploratory aim (*n *= 4). There are five different data collection techniques used in the publications reviewed. Studies used secondary data/patient records (*n *= 14), unstructured/semi-structured interviews (*n =* 5), observations (*n =* 2), online web-based questionnaires/assessments (*n* = 1) and standardized questionnaires/interviews/survey (*n* = 1). Studies targeted great variation of participants: patients with specific disease/condition (*n* = 11), older adults (*n* = 7), healthcare professionals (*n* = 6), social care specialists (*n =* 1) researchers (*n =* 1), policy-makers (*n =* 1) and patient’s family (*n =* 1). One study did not specify the study group [37]. Some studies (*n* = 8) targeted more than one group of participants simultaneously.

**Table 2:** Study characteristics

Article	Year of publication	Type of study	Research approach	Data collection	Study group	Quality of the study
**Anell & Glenngard (2014)** [26]	2014	Explan	Mixed	Unstructured/semi structured interviews + Secondary data/patient records	Healthcare professionals	Moderate
**Baumann et al., (2007)** [27]	2007	Explan	Qual	Unstructured/semi structured interviews	Healthcare professionals + Social care specialists + older adults	Moderate
**Birkmeyer et al., (2010)** [28]	2010	Explan	Quan	Secondary data/patient records	Older adults + with specific disease/condition	Low
**Busetto et al., (2017)** [29]	2017	Explor	Qual	Unstructured/semi structured interviews	Healthcare professionals	Moderate
**Briggs & Carvalho (2018)** [30]	2018	Explor	Qual	Online web based questionnaires/assessments	Healthcare professionals + Policy makers + Researchers	High
**Chen & Cheng (2016) **[31]	2016	Explan	Quan	Secondary data/patient records	Patients with specific disease/condition	Moderate
**Cheng, Lee & Chen (2012)** [32]	2012	Explan	Quan	Secondary data/patient records	Patients with specific disease/condition	Low
**Pan, Kung, Chiu, Liao & Tsai (2017)** [33]	2017	Explan	Quan	Secondary data/patient records	Patients with specific disease/condition	Moderate
**Ekdahl (2013) **[34]	2013	Explor	Mixed	Observations + Unstructured/semi structured interviews + Standardized questionnaires/interviews/surveys	Healthcare professionals + Older adults +	Moderate
**Fagan et al., (2010)** [35]	2010	Explan	Quan	Secondary data/patient records	Older adults + with specific disease/condition	Low
**Hollander & Kadiec (2015)** [36]	2015	Explan	Quan	Secondary data/patient records	Patients with specific disease/condition	Low
**Huitberg, Glendinning, Allebeck & Lönnroth (2005)** [37]	2005	Explan	Qual	Secondary data/patient records	No specific study group	Moderate
**Kasteridis et al., (2016)** [38]	2016	Explan	Quan	Secondary data/patient records	Older adults + with specific disease/condition	Low
**Kim et al., (2015)** [39]	2015	Explan	Quan	Secondary data/patient records	Patients with specific disease/condition	Low
**Laugaland, Aase & Waring (2014)** [40]	2014	Explor	Qual	Observations + Unstructured/semi structured interviews	Healthcare professionals + Older adults + family	High
**Nishi, Maeda & Babazono (2017)** [41]	2017	Explan	Quan	Secondary data/patient records	Older adults + with specific disease/condition	Low
**Nolan (2011)** [42]	2011	Explan	Quan	Secondary data/patient records	Older adults	Low
**Pizer & Gardner (2011)** [43]	2011	Explan	Quan	Secondary data/patient records	Patients with specific disease/condition	Low
**Yu, Tsai & Kung (2013)** [44]	2013	Explan	Quan	Secondary data/patient records	Patients with specific disease/condition	Moderate
**Total** **Number of studies shown in parentheses**	2018 (1) 2017 (3) 2016 (2) 2015 (2) 2014 (2) 2013 (2) 2012 (1) 2011 (2) 2010 (2) 2007 (1) 2005 (1)	Explan (15) Explor (4)	Quan (12) Qual (5) Mixed (2)	Secondary data/patient records (14) Unstructured/semi structured interviews (5) Observations (2) Online web based questionnaires/ Assessments (1) Standardized questionnaires/interviews/surveys (1)	Patients with specific disease/condition (11) Older adults (7) Healthcare professionals (6) Social care specialists (1) Researchers (1) Policy makers (1) Family (1) No specific study group (1)	Low (9) Moderate (8) High (2)

The sum of N per category can exceed 19 as papers can be classified into multiple sub-categories

*Note: Quan* Quantitative, *Qual* Qualitative, *Explan* Explanatory, *Explo* Exploratory

All publications included in this review focused specifically on financial incentives. Among the 19 studies selected for the review, nine studies discuss the role of rewards, six publications report on reimbursement mechanisms and three focused on penalties. Two studies do not report on any specific type of financial mechanism but instead stress, in general, the importance of appropriate financing mechanisms to improve care for older adults [30, 34].

We identify financial incentives that aim to improve care for patients with specific condition/disease (*n* = 8) and/or older adults (*n* = 7). Six studies do not report on financial incentives to have any specific target group.

These financial incentives are discussed with relation to various settings such as primary care (*n* = 12), hospital (*n* = 6) and social sector (*n* = 3). Two studies report on the use of financial incentives for all healthcare providers and other care providers in general [28, 37].

**Fig. 2:**
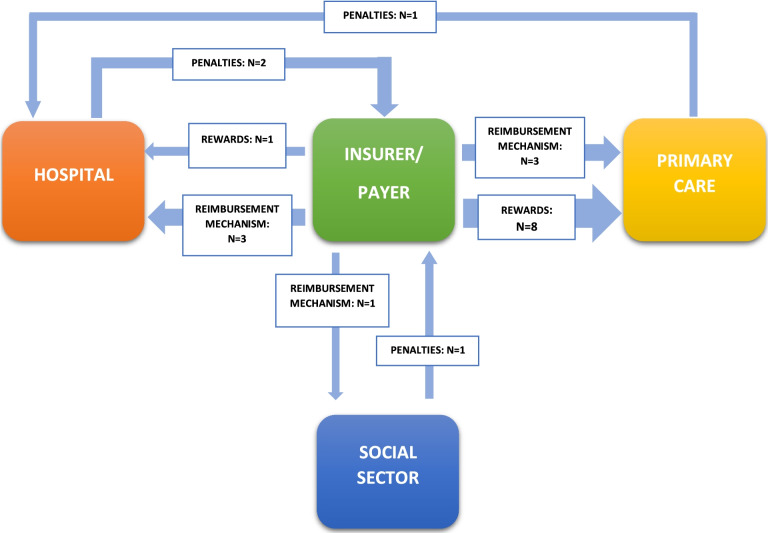
Financial incentives and settings identified in the literature (N indicates the number of publications found in the review)

Figure 2 presents the types of financial incentives and intervention settings that were identified in the literature. We identify 8 studies investigating the role of rewards in primary care and one study focusing on rewards in hospitals [41]. Reimbursement mechanisms are discussed with relation to primary care in three studies [28, 42, 43] and hospitals in three studies [28, 29, 39]. In addition, researchers are focusing on penalties in settings, such as hospital (*n* = 2) [27, 39], social sector [27] and primary care [40]. Three studies discuss financial incentives targeting simultaneously more than one setting, e.g. all care providers [27, 28, 37]. Two of those studies do not specify the setting but rather argue that the financial incentives target all (health) care providers [28, 37]. Two studies do not mention any setting [30, 34].

There is also great diversity with regard to the country where the intervention is reported. Some studies focus on the role of financial incentives in the United States (*n* = 5), Taiwan (*n* = 4), United Kingdom (*n* = 3), Sweden [26], Japan [41], Germany [29], Canada [36], Norway [40] and Ireland [42]. Two studies do not focus on any particular intervention country but rather discuss the importance of appropriate financial incentives [30, 34].

Quality assessment of included publications is also presented in Table 2. All publications, regardless of their quality, were included in the final analysis.

### Characteristics of Financial Incentives

Characteristics of financial mechanisms are presented in Table 3. The majority of the studies discuss the role of rewards and their impact on care coordination. For instance, providers may get rewarded for improving structure, outcome and process indicators [29, 33, 44] or for inter-provider care planning [41]. Most studies, 8 out of 9, discuss the role of P4P programs in rewarding healthcare providers [26, 31–33, 35, 36, 38, 44]. The study by Yu, Tsai & Kung (2013) [44] presents the P4P program for diabetes care implemented in Taiwan that provided financial incentives to medical care personnel for enhanced monitoring and subsequent care for patients along with a bonus for improved treatment outcomes. This program aims to increase the financial incentives for physicians to provide holistic care for diabetic patients, who might also be LTC users (study included high proportion of individuals 75 years and older). Similarly, Pan, Kung, Chiu, Liao & Tsai (2017) [33] report on financial incentives in the form of P4P program that reward healthcare providers for achieving pre-established criteria for treating specific diseases.

**Table 3:** Characteristics of financial incentives

Article	Type of financial incentive	Aim of the mechanism	Group targeted through mechanism	Intervention setting	Intervention country
**Anell & Glenngard (2014)** [26]	**REWARD** Pay-for-performance (use of outcome and process indicators)	No information provided	Older adults	Primary care	Sweden
**Baumann et al., (2007)** [27]	**PENALTY** For delays from hospital	Inter-agency collaboration ↑ Efficiency of discharge ↑ Discharge planning arrangements ↑	Older adults	Hospital + Social sector	United Kingdom
**Birkmeyer et al., (2010)** [28]	**REIMBURSMENT MECHANISM** Bundled payment for care around a surgical episode	Coordination of care ↑ Quality of care ↑ Cost-efficiency ↑	No specific requirement	Hospital + Primary care + other care providers	United States
**Busetto et al., (2017)** [29]	**REIMBURSMENT MECHANISM** G-DRG -For the integrated care intervention + obligatory number of treatment sessions	Effectiveness ↑ Efficiency ↑ Patient centeredness ↑ Satisfaction ↑ Safety ↑	Older adults	Hospital	Germany
**Briggs & Carvalho (2018)** [30]	Information not provided	Information not provided	Older adults	Information not provided	Information not provided
**Chen & Cheng (2016) **[31]	**REWARD** Pay-for-performance (For providing enhanced, guideline-based care)	Healthcare provision ↑ Continuity-of-care ↑ Healthcare outcomes ↑	With specific condition/disease	Primary care	Taiwan
**Cheng, Lee & Chen (2012)** [32]	**REWARD** Pay-for-performance (for 3 types of comprehensive visits)	Healthcare provision ↑	With specific condition/disease	Primary care	Taiwan
**Pan, Kung, Chiu, Liao & Tsai (2017)** [33]	**REWARD** Pay-for-performance (For improvement in 4 indicators, additional bonus for being the best)	Patient outcomes ↑ Quality of care ↑	With specific condition/disease	Primary care	Taiwan
**Ekdahl (2013) **[34]	Information not provided	Information not provided	Older adults	Information not provided	Information not provided
**Fagan et al., (2010)** [35]	**REWARD** Pay-for-performance (Bonus payment for meeting specific quality indicator goals)	Quality of care ↑ Resource use ↓	No specific requirement	Primary care	United States
**Hollander & Kadiec (2015)** [36]	**REWARD** Pay-for-performance (For providing enhanced, guideline-based care)	Annual healthcare costs ↓, Hospital utilization ↓	With specific condition/disease	Primary care	Canada
**Huitberg, Glendinning, Allebeck & Lönnroth (2005)** [37]	**REIMBURSMENT MECHANISM** Pooled budgets to integrate health and welfare services (social and other services)	Coordination ↑ Efficiency ↑ Flexibility in the use of resources ↑	No specific requirement	Health care providers + Social sector	United Kingdom
**Kasteridis et al., (2016)** [38]	**REWARD** Pay-for-performance (for identification and annual review of dementia patients)	Discharge process ↑	With specific condition/disease	Primary care	United Kingdom
**Kim et al., (2015)** [39]	**REIMBURSMENT MECHANISM** Prospective payments based on DRG – DRG-specific short stay threshold	Short-stays in the long-term care hospitals ↓ Unnecessary transfers ↓	requirement	Hospital	United States
	**PENALTY** For short stay under the threshold	Short-stays in the long-term care hospitals ↓ Unnecessary transfers ↓	No specific No specific requirement	Hospital	United States
**Laugaland, Aase & Waring (2014)** [40]	**PENALTY** For delayed discharge from the hospital	Patient flow ↑ Delayed discharge ↓	No specific requirement	Municipality (primary care)	Norway
**Nishi, Maeda & Babazono (2017)** [41]	**REWARD** For the inter-provider care-planning	Length of stay ↓ Total charge ↓	Older adults + with specific condition/disease	Hospital	Japan
**Nolan (2011)** [42]	**REIMBURSMENT MECHANISM** Eligibility for free primary care	Avoidable hospitalizations↓	Older adults	Primary care	Ireland
**Pizer & Gardner (2011)** [43]	**REIMBURSMENT MECHANISM** Fragmented financing	Continuity-of-care ↓ Health outcomes ↓	With specific condition/disease	Primary care	United States
**Yu, Tsai & Kung (2013)** [44]	**REWARD** Pay-for-performance (for improved health outcomes)	Holistic care ↑ Emergency department visits ↓	With specific condition/disease	Primary care	Taiwan
**Total** **Number of studies shown in parentheses**	Rewards (9) Reimbursement mechanism (6) Penalties (3)		With specific condition/disease (8) Older adults (7) No specific requirement (6)	Primary care (12) Hospital (6) Social sector (2) Health care providers (1) Other care providers (1)	United States (5) Taiwan (4) United Kingdom (3) Sweden (1) Japan (1) Germany (1) Canada (1) Norway (1) Ireland (1) Information not provided (2)

The sum of N per category can exceed 19 as papers can be classified into multiple sub-categories

↑ - increase, improve

↓ - decrease

In this program, quality performance is monitored by four indicators. Providers that score high in those indicators and are ranked at the top of their peers and are eligible for additional bonuses. This program motivates physicians to follow up with their patients. Another study discusses the role of a P4P program in which practices are given a bonus payment for meeting specific quality indicators [35]. Only one study focuses on reward in the form of additional “regional inter-provider care planning fee” [41]. In order to be eligible for this fee, providers have to plan disease-oriented clinical care pathways among different providers.

Moreover, researchers in publications discuss diverse reimbursement mechanisms. These reimbursement mechanisms refer to the fragmented financing and its impact on care coordination [43], an extension of eligibility for free primary care [42] and the use of pooled budgets to integrate health and welfare services [37]. Furthermore, studies address the use of bundled payments for care episodes [28] and “early complex rehabilitation” (mechanism) under German system of disease-related groups (G-DRG) [42]. Under “early complex rehabilitation” specific reimbursement system, geriatric hospitals in Germany receive bundled reimbursements for the treatment of similar groups of patients. These types of reimbursement are financially advantageous compared to the regular rates. Geriatric hospitals are eligible for it if they provide integrated care intervention and obligatory number of treatment sessions. Study by Birkmeyer and colleagues (2010) [28] also discusses bundled payments, but for care around a surgical episode for following procedures: coronary artery bypass, hip fracture repair, back surgery and colectomy - procedures common among LTC users. Participants had to be 65 years and older to be included in the study. Bundling entails lumping reimbursements to healthcare and other care providers into a single payment. The primary motivation underlying bundled payments is improving care coordination, quality of care and cost-effectiveness.

Besides rewards and reimbursement mechanisms, in this review, we identify penalties that are issued with relation to patient discharge, for either delayed [27, 40] or too-early discharge before the patient is medically stable enough to go home [39]. Penalties for delayed hospital discharges of older adults aim to stimulate a good patient flow between care providers and to overcome challenges with delayed discharges. Studies on penalties included in our review focus on older adults that may be in need of LTC. A study by Laugaland, Aase & Waring (2014) [40] elaborates on penalties that have to be paid to an acute provider unit (533 euros per day) by the municipality in a situation when ready for discharge patient is not accepted on time. This particular type of penalty incentivize discharge planning and encourages coordination. On the other hand, Kim et al. (2015) [39] studied the use of penalties for a short stay (too-early discharge) under the threshold in LTC hospitals. Through this penalty, providers were encouraged to keep the patients until after their lengths-of-stay have exceeded the short-stay threshold.

### Impact of the Financial Incentives

As shown in Table 4, majority of studies (*n* = 16) investigate the impact of the financial incentives on care coordination that is measured with the use of process and/or outcome indicators. Three studies do not measure the effect of financial incentives [26, 30, 34]. Overall, from included studies, seven studies report on the positive effect of financial incentives on care coordination, six studies demonstrate to have unclear or have no effect, and three studies show a negative effect of financial incentives. In general, the study outcomes are heterogeneous, thus difficult to compare. A detailed description of outcomes can be found in additional file (see Supplementary Table 2).

**Table 4:** Financial incentives – impact on measured indicators

Article	Financial incentives	Measurement	Impact on measured indicators
**Anell & Glenngard (2014)** [26]	P4P	Utilization of hospital care, number of bed-days	Information not provided
**Baumann et al., (2007)** [27]	Penalties for delayed discharge for responsible party	Information not provided	+
**Birkmeyer et al., (2010)** [28]	Episode-based payment bundling, single payment to all providers for care around surgical episode	Average total payments around inpatient surgery (hospital, physician, post-acute care) 30 days readmission	+/−
**Busetto et al., (2017)** [29]	Early complex geriatric rehabilitation	Effectiveness, efficiency, patient-centeredness, satisfaction, safety	–
**Briggs & Carvalho (2018)** [30]	Information not provided	Information not provided	Information not provided
**Chen & Cheng (2016) **[31]	P4P	The number of essential examinations/tests, continuity of care, health care outcomes	+
**Cheng, Lee & Chen (2012)** [32]	P4P	Long-term effects of P4P program, healthcare utilization - Essential examinations/tests performed at diabetes-related physician visits, Diabetes-related hospitalizations, Diabetes-related health care expenses Impact on overall health care expenses, including both diabetes-related and nondiabetic-related conditions.	+
**Pan, Kung, Chiu, Liao & Tsai (2017)** [33]	P4P	Mortality, patients’ physician continuity	+
**Ekdahl (2013) **[34]	Information not provided	Information not provided	Information not provided
**Fagan et al., (2010)** [35]	P4P	Quality of care for the incentivized care indicators, quality of care for the nonincentivized care indicators, utilization and medical costs incurred	+/−
**Hollander & Kadiec (2015)** [36]	P4P	Total annual costs of health care, number of indicators of hospital utilization	+
**Huitberg, Glendinning, Allebeck & Lönnroth (2005)** [37]	Pooled budgets to integrate health and welfare services	Coordination Cost-effectiveness Experiences of service users	+/−
**Kasteridis et al., (2016)** [38]	P4P	Likelihood of care home placement following acute hospital admission	+
**Kim et al., (2015)** [39]	DRG-specific short-stay threshold	Information not provided	–
**Laugaland, Aase & Waring (2014)** [40]	Penalties for delayed discharge	Information not provided	–
**Nishi, Maeda & Babazono (2017)** [41]	Regional inter-provider care-planning fee	LOS, total charge	+/−
**Nolan (2011)** [42]	Eligibility for free primary care	Avoidable hospitalizations	+/−
**Pizer & Gardner (2011)** [43]	Fragmented financing	Hospitalizations for ambulatory care sensitive conditions	+
**Yu, Tsai & Kung (2013)** [44]	P4P	Emergency department visits	+/−

+ improved

+/− no effect or effect unclear

- negative effect

/ lack of data

Studies on financial rewards provide mixed results. For instance, Hollander & Kadiec (2015) [36] show that the use of rewards related to care transition can and do avoid costs for the health care system and reduce hospital utilization. Study reported on four conditions that are common among geriatric patients: diabetes, coronary heart failure, congestive pulmonary disease, and hypertension. The study of Chen & Cheng (2016) [31] and Cheng, Lee & Chen (2012) [32] reports that rewards in the form of P4P program might lead to better care continuity and ultimately decrease the likelihood of hospital admissions or emergency department (ED) visits. Nonetheless, studies by Fagan and colleagues (2010) [35] and Yu, Tsai & Kung (2013) [44] found no evidence on P4P programs to improve quality of care and resource use.

Furthermore, studies on the use of penalties also provide inconsistent results. The study of Baumann et al. (2007) [27] argues that penalties for delayed discharge increase the efficiency of collaboration with social services and enhance the use of integrated discharge planning teams. In contrast, the study carried by Laugaland, Aase & Waring (2014) [40] shows that penalties may also have a negative impact on care transition. Penalties may result in providers rushing patient transfers.

Similar to other financial incentives, we also observe mixed results in the studies on reimbursement mechanisms. For instance, Nolan (2011) [42] observe no change in the number of avoidable hospitalizations, as a result of a reimbursement mechanism that extended eligibility for primary care for older adults. Furthermore, contrary to some assumptions, the study by Huitberg, Glendinning, Allebeck & Lönnroth (2005) [37] argues that pooled budgets between healthcare and the social sector have no impact on cost-effectiveness, the behavior of front-line professionals and experiences of service users. On the other hand, the study by Busetto and colleagues (2017) [29] carried out in geriatric hospital focus on patients with complex, multiple age-related conditions that require long-term care after discharge. The study reports that the use of bundled payments with an obligatory number of treatment sessions may lead to the “revolving door effect”, unnecessary incurrence of costs (efficiency), an increased likelihood of adverse events or medical mistakes.

## Discussion

To our knowledge, this is the first study that presents evidence on financial aspects that affect care transition of older adults in LTC systems. We are also first to identify the settings in which these financial aspects play a significant role. Moreover, we synthesize the reported impact of these financial aspects on care coordination/care transition. We included 19 studies in this review.

We found that financial aspects and specifically financial incentives may play an important role in the LTC systems by either improving or hampering care transitions of older adults. Our findings that financial incentives may play an important role in the way healthcare is provided are in line with assumptions coming from microeconomic theory [13], the theory of principal agent-behavior [14], and behavioral economics [15]. These assumptions assume that financial incentives are likely to influence providers’ behavior. Furthermore, researchers also point out the importance of financial incentives in stimulating the integration of care [9, 45, 46]. For instance, a study by Struckmann, Quentin, Busse & van Ginneken, (2017) [46] suggests that innovative payment mechanisms, such as P4P and pay for coordination (P4C) have the potential to encourage providers to collaborate and improve care delivery process.

We identified three types of financial incentives that may play a significant role in care transition and care coordination as a whole. These financial incentives involve reimbursement mechanism, reward, and penalty. This is not surprising as monetary incentives that stimulate the integration of providers and promote effective chronic care have been an issue of debate for researchers worldwide [9, 46, 47]. In economic theory, financial incentives may lead to behavior change of providers, patients and other stakeholders and thus, stimulate immediate and long-term improvements in performance [9, 48]. Different techniques for financing providers have implications on the nature and quality of services provided [9]. For instance, paying each care provider involved in the care transition separately does not incentivize the providers to coordinate the care and may even block effective integration [11, 46]. Thus, alternative approaches of provider payment mechanisms, such as P4C, P4P, Pay for Quality (P4Q), bundled payments and shared-savings models etc., may encourage the integration of providers to work together towards coordinated care [9, 46]. These innovative payment mechanisms allow to offset the inherent limitations of traditional payment methods and stimulate providers to provide high-quality care by rewarding collaboration with different stakeholders. Improved collaboration between different professions, organizations and sectors is especially important during transitions of care.

Beside the crucial role of reimbursement mechanisms and rewards to stimulate integration of care, studies included in our review also discussed the role of penalties. Providers could be penalized for poor performance, particularly with regard to poor discharge planning. In theory, penalties may alter providers’ efforts to improve quality of care. According to Dickinson (2001) [49], penalties may create an even stronger providers’ response than rewards of equivalent size due to risk aversion or” loss aversion”. Nevertheless, in practice, it is not always the case, as it was demonstrated in the reviewed studies. In addition, their fairness and likelihood of driving appropriate behavior are still debated [3].

Furthermore, we found that the highest interest in financial incentives was in primary care settings. According to the report of WHO (2016) [1], rewarding primary care doctors for their efforts in coordinating care is an important aspect motivating them to follow up with the patient. It is crucial because primary care physicians (PCPs) are patients’ first point of contact and their service has an overwhelming bearing on healthcare quality. Moreover, they are often crucial players in coordinating services delivered by different stakeholders [50, 51]. PCPs play an important role not only for LTC patients that are at home but also for the ones residing in nursing facilities. A study by Codde, Frankel, Arendts and Babich (2010) [52] found that 31% of all emergency department visits from residential aged care facilities could be avoided with improved primary care. PCPs are also important actors when it comes to identifying risks among frail older adults and preventing hospitalizations. Their responsibility is to detect high-risk patients and refer them to appropriate care and treatment [53]. As a result, application of financial incentives in primary care that directly reward “performance” and “quality” is gaining recognition worldwide and this was reflected in the studies that we included in our analysis. Our review found that especially P4P programs are common to reward high-performing primary care physicians. These programs rewarded improvement in structure, outcome and process indicators. Nevertheless, the effects of the P4P scheme remain largely uncertain [54]. Two separate studies carried out by Mendelson et al. (2017) [55] and Langdown & Peckham (2014) [56] suggest that P4P programs offer only short-term improvements and have no impact on long-term patient outcomes.

Majority of studies included in this review measure the impact of reported financial incentives on predetermined indicators. Nonetheless, drawing one single conclusion on the impact of these financial incentives on care transition in LTC systems seem infeasible. This is due to the heterogeneity of studied financial incentives, settings in which they are applied and their intermediate goals. Moreover, studies focus on financial incentives in their specific contexts and national health systems in which they operate. Perhaps, financial incentives improving care coordination and care transition in one country may not have the same effect in another [46]. Therefore, prior to implementation, financial incentives should be developed and tailored to the local context.

We need to emphasize that measuring indicators and outcomes in LTC can be problematic, and quality can be difficult to define. First, the concept of LTC quality is multifaceted. Up to date, there is no definition of what constitutes LTC quality [57]. Additionally, measuring some of the indicators may be very challenging. Collection of data on LTC quality also poses a lot of challenges. Many countries do not measure outputs but instead, collect data on inputs such as the number of beds in nursing homes. Second, patient information of diagnosis, functional status, and medical complexity is usually not available. Even if such information would be available, there is a methodological challenge related to the focus of quality in LTC. Majority of individuals in need of LTC are older adults and their autonomy is likely to worsen with age. Thus, the main focus of quality in LTC settings is to some extent reduce dependency and disability by helping dependent individuals to maintain control over their condition. Defining a start and end point for measurement in LTC may be also problematic. Third, LTC recipients often navigate across care settings which further complicate the measurement of LTC quality [53, 57]. There are also other non-medical factors such as housing and adaptation of the environment for the people with disabilities that may affect the LTC quality. Taking into consideration all these aspects, it remains a challenge to evaluate the impact of financial incentives on LTC quality. Thus, the first step is to develop a set of standardized indicators that would capture the nature of LTC and implement it into practice.

We need to acknowledge that some of the examples of diseases and conditions in the included studies do not seem to refer to classical LTC users. Nonetheless, conditions such as diabetes and hypertension etc. most commonly develop in older adults and have a high prevalence in LTC facilities and, in general, LTC users. Diabetes in senior patients is often associated with limitations in physical function and disability and may increase the likelihood of institutionalization. For diabetic older adults care transitions are very common and these patients are particularly at high risk of adverse events. Thus, diabetes management in older adults is crucial to optimize care transition [58]. This applies to other chronic conditions as well. If not managed properly on time, chronic diseases in LTC users may lead to hospitalization, irreversible deterioration and increased dependency.

### Limitations and recommendations for future research

Our study has some limitations. First, the research string build for this review might not have identified all relevant literature on financial aspects that affect care transition. This is mainly due to the heterogeneity of terminology for transitional care. It is noteworthy that the terms “transitional care” and “care transition” are still not widely used by researchers. In the included studies, authors often refer to continuity of care, care coordination and integrated care instead. Furthermore, studies included in this review had diverse research designs and focused on different financial mechanisms, care settings, outcome measurements and countries. We also recognize possible publication bias since some relevant papers might have been under review, not yet published, or published in grey literature sources, which we did not review. We also acknowledge possible selection bias even though a part of the selection process was verified by other researchers in the team.

On the other hand, we tried to mitigate selection and publication bias by a rigorous systematic review of published and unpublished studies. We contacted all authors of studies that were unavailable online and requested full-text. Moreover, we considered all studies independently of the language.

### Practice and/or Policy Implications

Well-developed and tailored financial incentives have the potential to stimulate care coordination and improve care transitions for older patients in LTC systems. Policy-makers should consider the implementation of different financial incentives such as reimbursement mechanisms, rewards and penalties among care providers to improve care transitions among older adults. Once implemented, new financing mechanisms should be continuously evaluated to inform future policy.

Beyond identifying financial aspects, and particularly financial incentives, that have an impact on care transition, there is a need to examine the effect of these various monetary incentives. Future studies that focus on evaluating the effects of financial incentives should perform age stratification in their data sets. This would enable us to observe the impact and the extent of the financial incentive among different age groups, particularly older adults.

Moreover, to our knowledge, as indicated in this review, there are no studies that discuss how the financing of LTC systems affects the direction of the transition. Perhaps older adults will be more likely to be institutionalized despite their ability and willingness to stay at home? We hypothesize that the way LTC systems are financed will have implications on the direction of the transition. Therefore, future studies should explore the link between these two variables.

## Conclusions

Overall, our results suggest that financial incentives are potentially powerful tools to improve care transition among older adults in LTC systems. In this review, we identified three types of financial incentives that may play a significant role in care transition, respectively, reimbursement mechanism, reward and penalty. In addition, we found that the highest interest in financial incentives was in primary care settings. However, given the diversity of the studies, we are unable to draw firm conclusions regarding the impact of these financial incentives on care transition in LTC system. In this regard, more evidence of the impact of monetary incentives on care transition among older adults is needed. In particular, it is imperative that future research investigates the causality of this relationship to be able to support the improvement of care transition.

## Supplementary Information

**Additional file 1: Supplementary Table 1**. PRISMA 2020 Checklist. **Supplementary Table 2**. Financial mechanisms – outcomes and recommendations.

**Additional file 2: Appendix 1.**

## Data Availability

All relevant data are within the paper and its Supporting Information files.

## Abbreviations

CASP: Critical Appraisal Skills Programme
EPHPP: Effective Public Health Practice Project
G-DRG: Disease Related Groups
LTC: Long-Term Care
PCPs: Primary Care Physicians
P4C: Pay For Coordination
P4P: Pay For Performance
P4Q: Pay For Quality
