# Supplementary material for: Do financial aspects affect care transitions in long-term care systems? A systematic review
Source: Arch Public Health. 2022 Mar 23;80:90. doi: 10.1186/s13690-022-00829-y (PMC8941782; doi:10.1186/s13690-022-00829-y)
Supplement: Supplementary file 1 — Additional file 1: Supplementary Table 1. PRISMA 2020 Checklist. Supplementary Table 2. Financial mechanisms – outcomes and recommendations. [file 13690_2022_829_MOESM1_ESM.docx]

## Supplementary material

**Supplementary Table 1**. PRISMA 2020 Checklist

| **Section and Topic** | **Item #** | **Checklist item** | **Location where item is reported** |
| --- | --- | --- | --- |
| **TITLE** | | |  |
| Title | 1 | Identify the report as a systematic review. | 6 |
| **ABSTRACT** | | |  |
| Abstract | 2 | See the PRISMA 2020 for Abstracts checklist. | Done |
| **INTRODUCTION** | | |  |
| Rationale | 3 | Describe the rationale for the review in the context of existing knowledge. | 4-6 |
| Objectives | 4 | Provide an explicit statement of the objective(s) or question(s) the review addresses. | 6 |
| **METHODS** | | |  |
| Eligibility criteria | 5 | Specify the inclusion and exclusion criteria for the review and how studies were grouped for the syntheses. | 7-10 |
| Information sources | 6 | Specify all databases, registers, websites, organisations, reference lists and other sources searched or consulted to identify studies. Specify the date when each source was last searched or consulted. | 7 |
| Search strategy | 7 | Present the full search strategies for all databases, registers and websites, including any filters and limits used. | 7/Appendix |
| Selection process | 8 | Specify the methods used to decide whether a study met the inclusion criteria of the review, including how many reviewers screened each record and each report retrieved, whether they worked independently, and if applicable, details of automation tools used in the process. | 7-8 |
| Data collection process | 9 | Specify the methods used to collect data from reports, including how many reviewers collected data from each report, whether they worked independently, any processes for obtaining or confirming data from study investigators, and if applicable, details of automation tools used in the process. | 8 |
| Data items | 10a | List and define all outcomes for which data were sought. Specify whether all results that were compatible with each outcome domain in each study were sought (e.g. for all measures, time points, analyses), and if not, the methods used to decide which results to collect. | Not applicable |
|  | 10b | List and define all other variables for which data were sought (e.g. participant and intervention characteristics, funding sources). Describe any assumptions made about any missing or unclear information. | Not applicable |
| Study risk of bias assessment | 11 | Specify the methods used to assess risk of bias in the included studies, including details of the tool(s) used, how many reviewers assessed each study and whether they worked independently, and if applicable, details of automation tools used in the process. | 9 |
| Effect measures | 12 | Specify for each outcome the effect measure(s) (e.g. risk ratio, mean difference) used in the synthesis or presentation of results. | Not applicable |
| Synthesis methods | 13a | Describe the processes used to decide which studies were eligible for each synthesis (e.g. tabulating the study intervention characteristics and comparing against the planned groups for each synthesis (item #5)). | Not applicable |
|  | 13b | Describe any methods required to prepare the data for presentation or synthesis, such as handling of missing summary statistics, or data conversions. | 9-10 |
|  | 13c | Describe any methods used to tabulate or visually display results of individual studies and syntheses. | 9-10 |
|  | 13d | Describe any methods used to synthesize results and provide a rationale for the choice(s). If meta-analysis was performed, describe the model(s), method(s) to identify the presence and extent of statistical heterogeneity, and software package(s) used. | Partially explained at pages 9-10 |
|  | 13e | Describe any methods used to explore possible causes of heterogeneity among study results (e.g. subgroup analysis, meta-regression). | Not applicable |
|  | 13f | Describe any sensitivity analyses conducted to assess robustness of the synthesized results. | Not applicable |
| Reporting bias assessment | 14 | Describe any methods used to assess risk of bias due to missing results in a synthesis (arising from reporting biases). | Not applicable |
| Certainty assessment | 15 | Describe any methods used to assess certainty (or confidence) in the body of evidence for an outcome. | Not applicable |
| **RESULTS** | | |  |
| Study selection | 16a | Describe the results of the search and selection process, from the number of records identified in the search to the number of studies included in the review, ideally using a flow diagram. | 10 |
|  | 16b | Cite studies that might appear to meet the inclusion criteria, but which were excluded, and explain why they were excluded. | PRISMA flowchart |
| Study characteristics | 17 | Cite each included study and present its characteristics. | Throughout the result section |
| Risk of bias in studies | 18 | Present assessments of risk of bias for each included study. | 25-26 |
| Results of individual studies | 19 | For all outcomes, present, for each study: (a) summary statistics for each group (where appropriate) and (b) an effect estimate and its precision (e.g. confidence/credible interval), ideally using structured tables or plots. | Not applicable |
| Results of syntheses | 20a | For each synthesis, briefly summarise the characteristics and risk of bias among contributing studies. | Not applicable |
|  | 20b | Present results of all statistical syntheses conducted. If meta-analysis was done, present for each the summary estimate and its precision (e.g. confidence/credible interval) and measures of statistical heterogeneity. If comparing groups, describe the direction of the effect. | Not applicable |
|  | 20c | Present results of all investigations of possible causes of heterogeneity among study results. | Not applicable |
|  | 20d | Present results of all sensitivity analyses conducted to assess the robustness of the synthesized results. | Not applicable |
| Reporting biases | 21 | Present assessments of risk of bias due to missing results (arising from reporting biases) for each synthesis assessed. | Not applicable |
| Certainty of evidence | 22 | Present assessments of certainty (or confidence) in the body of evidence for each outcome assessed. | Not applicable |
| **DISCUSSION** | | |  |
| Discussion | 23a | Provide a general interpretation of the results in the context of other evidence. | 15-19 |
|  | 23b | Discuss any limitations of the evidence included in the review. | 18-19 |
|  | 23c | Discuss any limitations of the review processes used. | 18-19 |
|  | 23d | Discuss implications of the results for practice, policy, and future research. | 19 |
| **OTHER INFORMATION** | | |  |
| Registration and protocol | 24a | Provide registration information for the review, including register name and registration number, or state that the review was not registered. | 6 |
|  | 24b | Indicate where the review protocol can be accessed, or state that a protocol was not prepared. | 6 |
|  | 24c | Describe and explain any amendments to information provided at registration or in the protocol. | Not applicable |
| Support | 25 | Describe sources of financial or non-financial support for the review, and the role of the funders or sponsors in the review. | 6, 20 |
| Competing interests | 26 | Declare any competing interests of review authors. | Not applicable |
| Availability of data, code and other materials | 27 | Report which of the following are publicly available and where they can be found: template data collection forms; data extracted from included studies; data used for all analyses; analytic code; any other materials used in the review. | Reported throughout the article |

**Supplementary Table 2**. Financial mechanisms – outcomes and recommendations

| Article | Financial mechanism | Measurement | Results | Recommendation | Impact on measured indicators |
| --- | --- | --- | --- | --- | --- |
| Anell & Glenngard (2014) | P4P | Utilization of hospital care, number of bed-days | There are certain problems associated with the process and outcome indicators in the primary care. Rewards may be random and do not reflect the actual engagement. | Authors suggestion is to use indicators for diagnostic purposes. Moreover, the use of indicators are most effective when integrated into a system of continuous monitoring and improvements. | Information not provided |
| Baumann et al., (2007) | Penalties for delayed discharge for responsible party | Information not provided | The efficiency of social services had improved. It also lead to early discharge schemes, increased use of nurse-led discharge and integrated discharge planning teams. | Authors suggest further research in ‘high performing’ sites as it should seek to examine the experiences of discharges patients. | + |
| Birkmeyer et al., (2010) | Episode-based payment bundling, single payment to all providers for care around surgical episode | Average total payments around inpatient surgery (hospital, physician, post-acute care)  30 days readmission | Average total payments for inpatient surgery episodes varied. Hospital payments accounted for the largest share of total payments. No evidence regarding savings achieved by episode-based bundled payments for inpatient surgery. Among specific types of payments, those associated with 30-day readmissions and postacute care varied most substantially across hospitals. | Fully bundled payments for inpatient surgical episodes would need to be dispersed among many different types of providers. There is a need for safeguards against unintended consequences of bundled payments. Bundled payments may provide incentives for hospitals and providers to skimp on necessary care. Hospitals should implement better systems for monitoring, benchmarking, and improving their quality and cost-efficiency with inpatient surgery. | +/- |
| Busetto et al., (2017) | Early complex geriatric rehabilitation | Effectiveness, efficiency, patient-centeredness, satisfaction, safety | Overuse of services by some patients, and under- and misuse of services by others, regardless of outcome. Revolving door effect. GFK contributed to unnecessary incurrence of costs (efficiency), an increased likelihood of adverse events or medical mistakes (safety), and frustration among staff (satisfaction). | Authors recommend and increased focus on trying to understand how intervention components interact with context factors and/ combined, lead to positive and/or negative outcomes. | - |
| Briggs & Carvalho (2018) | Information not provided | Information not provided | Information not provided | Information not provided | Information not provided |
| Chen & Cheng (2016) | P4P | The number of essential examinations/tests, continuity of care, health care outcomes | P4P program led to increase in the number of necessary examinations/tests and improved continuity of care between patients and their physicians, reduced likelihood of hospital admissions or ED visits. | Health authorities could develop policies to increase participation in P4P program and encourage continued improvement in health care outcomes. Moreover, it is recommended to investigate the reasons for inconsistent findings concerning the impact of P4P program in different health care systems for future studies. | + |
| Cheng, Lee & Chen (2012) | P4P | Long-term effects of P4P program, healthcare utilization - Essential examinations/tests performed at diabetes-related physician visits,  Diabetes-related hospitalizations,  Diabetes-related health care expenses  Impact on overall health care expenses, including both diabetes-related and nondiabetic-related conditions. | P4P patients had significantly more diabetes specific examinations and tests. They also had a significantly higher number of diabetes-related physician visits (1^st^ year) and had fewer diabetes-related hospitalization in the follow-up period. Health care expenses were higher for intervention group in the first year but spent significantly less than their counterparts in the subsequent years. | Authors suggest that this program could provide long-term cost savings for continual enrollees. Additional research is required to evaluate the cost-effectiveness of similar P4P programs. | + |
| Pan, Kung, Chiu, Liao & Tsai, 2017 (2017) | P4P | Mortality, patients’ physician continuity | Diabetes P4P participants have higher physician continuity and lower HR of mortality. P4P could increase survival or improve treatment outcomes. | Health policy makers should evaluate the possibility of P4P programs in treating other chronic diseases, such as hypertension or kidney kidney disease. | + |
| Ekdahl (2013) | Information not provided | Information not provided | Information not provided | It is essential to have remuneration system with incentives for continuity of care and which measures health outcomes other than those used for usual care e.g. quality of life and independence. | Information not provided |
| Fagan et al., (2010) | P4P | Quality of care for the incentivized care indicators, quality of care for the nonincentivized care indicators, utilization and medical costs incurred | This study did not generate evidence that P4P improved quality of care or resource use. | Authors suggest that perhaps further research involving interventions with clearer coordinator roles and/or larger financial incentives for physicians may demonstrate an improved intervention effect. | +/- |
| Hollander & Kadiec (2015) | P4P | Total annual costs of health care, number of indicators of hospital utilization | Incentive payments can and do avoid costs for the health care system (of course it depends which costs and which chronic conditions are looked at) and in general reduce patients’ utilization of more costly hospital services. | Information not provided | + |
| Huitberg, Glendinning, Allebeck & Lönnroth (2005) | Pooled budgets to integrate health and welfare services | Coordination  Cost-effectiveness  Experiences of service users | Limited evidence on the impact of pooled budgets on cost-effectiveness, behavior of front-line professionals, experiences of service users in England and Sweden. Creating and managing pooled budgets proved costly. | Additional work may also be required to help specific groups of professionals to break down their traditional differences in culture and ways of working. Need for major change management processes. Pooled budgets may be necessary, but not sufficient, factor in promoting service integration. | +/- |
| Kateridis et al., (2016) | P4P | Likelihood of care home placement following acute hospital admission | The review may reduce the probability of institutionalized care in those with dementia. | Information not provided | + |
| Kim et al., (2015) | DRG-specific short-stay threshold | Information not provided | Short-stay payment policy creates strong incentives for the hospitals to delay discharges until the threshold has been met. | There is urgent need to reexamine and restructure the payment system for long-term care hospitals and to clarify their expected and actual roles in care continuum, with the goal that incentives are properly aligned. | - |
| Laugaland, Aase & Waring (2014) | Penalties for delayed discharge | Information not provided | Some discharges have to be rushed or transferred directly to home, even if short-term nursing home stay was recommended – implications for the patients’ outcomes. | Given the interdependence among the functions, there is a need for corresponding multi-factorial interventions. Future research should focus on understanding the relationships between various functions and PSFs and their impact on hospital discharge practices and outcomes. | - |
| Nishi, Maeda & Babazono (2017) | Regional inter-provider care-planning fee | LOS, total charge | With the coordinated care group in the present study, we did not observe lower indices of healthcare resource utilization other than LOS in acute care. A payment system for care coordination is inappropriate. | Healthcare system reform is necessary to improve care continuity across multiple healthcare institutions in Japan. Payments should not be made separately to individual institutions and without an evaluation system. | +/- |
| Nolan (2011) | Eligibility for free primary care | Avoidable hospitalizations | Avoidable hospitalizations for the over 70s did declined but it also declined for the under 70s, meaning that a significant difference-in-difference effect could not be identified. | Information not provided | +/- |
| Pizer & Gardner (2011) | Fragmented financing | Hospitalizations for ambulatory care sensitive conditions | Fragmented financing has a statistically significant effect on the probability of ACSC hospitalization, the magnitude of this effect is relatively large. | Policy initiatives to reduce fragmented financing are warranted. | + |
| Yu, Tsai & Kung (2013) | P4P | Emergency department visits | The risk of emergency departments visits for diabetic hypoglycemia was higher in P4P-enroled patients. Emergency visits due to diabetic hypoglycemia after P4P were significantly higher than those before P4P. | Efforts should be made to increase health education or to improve monitoring of patients when caring for P4P patients. | +/- |
