# Supplementary material for: Do financial aspects affect care transitions in long-term care systems? A systematic review
Source: Arch Public Health. 2022 Mar 23;80:90. doi: 10.1186/s13690-022-00829-y (PMC8941782; doi:10.1186/s13690-022-00829-y)
Supplement: Supplementary file 2 — Additional file 2: Appendix 1. [file 13690_2022_829_MOESM2_ESM.docx]

Appendix 1

EMBASE

'aged'/exp OR aged:ab, ti OR aging:ab, ti OR elderly:ab, ti OR old:ab, ti OR senior*:ab, ti OR geriatric:ab, ti AND 'patient handoff' OR 'patient handover' OR 'hospital discharge'/exp OR 'patient transfer' OR 'transitional care'/exp OR 'clinical handover'/exp OR 'coordinated care'/exp OR 'coordination of care' OR 'care coordination' OR 'integrated care' OR 'patient care'/exp OR 'care continuum' AND 'financial management'/exp OR 'organization'/exp OR 'provision' OR 'purchasing'/exp OR 'reimbursement'/exp

PubMed

(“aged” [MeSH Terms] OR “aged” [Title/Abstract] OR “aging” [Title/Abstract] OR “elderly” [Title/Abstract] OR “old” [Title/Abstract] OR “senior*” [Title/Abstract] OR “geriatric” [Title/Abstract]) AND (“patient handoff” [MeSH Terms] or “patient handoff” [All Fields] OR “patient handover” [All Fields] OR “patient discharge” [MeSH Terms] OR “patient discharge” [All Fields] OR “patient transfer” [MeSH Terms] OR “Patient transfer” [All Fields] OR “transitional care” [MeSH Terms] OR “transitional care” [All Fields] OR “clinical handover” [All Fields] OR “coordinated care” [All Fields] OR “coordination of care” [All Fields] OR “care coordination” [All Fields] OR “integrated care” [All Fields] OR “care continuity” [All Fields] OR “continuity of care” [All Fields] OR “care continuum” [All Fields]) AND (“financing” [All Fields] OR “financ*” [All Fields] OR "funding" [All Fields] OR “organised” [All Fields] OR “organized” [All Fields] OR “organisational” [All Fields] OR “organizational” [All Fields] OR “organizing” [All Fields] OR “organising” [All Fields] OR “organization” [All Fields] OR “organisation” [All Fields] OR “provision” [All Fields] OR purchasing [All Fields] OR purchase* [All Fields] OR “reimbursement” [All Fields])

CINAHL

((MM "aged") OR (TI "aged") OR (AB "aged") OR (TI "aging") OR (AB "aging") OR (TI "elderly") (AB "elderly") OR (TI "old") OR (AB "old") OR (TI "senior*") OR (AB "senior*") OR (TI "geriatric") OR (AB "geriatric")) AND ((MM "hand off") OR (TX "hand off") OR (TX "patient handover") OR (MM "patient discharge") OR (TX "patient discharge") OR (TX "patient transfer") OR (MM "transitional care") OR (TX "transitional care") OR (TX "clinical handover") OR (TX "coordinated care") OR (TX "coordination of care") OR (TX "care coordination") OR (TX "integrated care") OR (MM "continuity of patient care") OR (TX "continuity of patient care") OR (TX "care continuum")) AND ((MM "financing, organized") OR (TX "financing") OR (TX "financ*") OR (TX "organi*ed") OR (TX "organi*ational") OR (TX "organi*ing") OR (TX "organi*ation") OR (TX "provision") OR (TX "purchasing") OR (TX "purchase*") OR (MM "reimbursement mechanism") OR (MM "reimbursement mechanism") OR (TX "reimbursement mechanism") OR (TX "reimbursement"))
